# IL-33 Drives Augmented Responses to Ozone in Obese Mice

**DOI:** 10.1289/EHP272

**Published:** 2016-07-29

**Authors:** Joel A. Mathews, Nandini Krishnamoorthy, David Itiro Kasahara, Youngji Cho, Allison Patricia Wurmbrand, Luiza Ribeiro, Dirk Smith, Dale Umetsu, Bruce D. Levy, Stephanie Ann Shore

**Affiliations:** 1 Department of Environmental Health, Harvard T.H. Chan School of Public Health, Boston, Massachusetts, USA; 2 Pulmonary and Critical Care Medicine, Harvard Institutes of Medicine Building, Boston, Massachusetts, USA; 3 Department of Inflammation Research, Amgen, Seattle, Washington, USA; 4 Genentech, South San Francisco, California, USA

## Abstract

**Background:**

Ozone increases IL-33 in the lungs, and obesity augments the pulmonary effects of acute ozone exposure.

**Objectives:**

We assessed the role of IL-33 in the augmented effects of ozone observed in obese mice.

**Methods:**

Lean wildtype and obese db/db mice were pretreated with antibodies blocking the IL-33 receptor, ST2, and then exposed to ozone (2 ppm for 3 hr). Airway responsiveness was assessed, bronchoalveolar lavage (BAL) was performed, and lung cells harvested for flow cytometry 24 hr later. Effects of ozone were also assessed in obese and lean mice deficient in γδ T cells and their wildtype controls.

**Results:**

and Discussion: Ozone caused greater increases in BAL IL-33, neutrophils, and airway responsiveness in obese than lean mice. Anti-ST2 reduced ozone-induced airway hyperresponsiveness and inflammation in obese mice but had no effect in lean mice. Obesity also augmented ozone-induced increases in BAL CXCL1 and IL-6, and in BAL type 2 cytokines, whereas anti-ST2 treatment reduced these cytokines. In obese mice, ozone increased lung IL-13+ innate lymphoid cells type 2 (ILC2) and IL-13+ γδ T cells. Ozone increased ST2+ γδ T cells, indicating that these cells can be targets of IL-33, and γδ T cell deficiency reduced obesity-related increases in the response to ozone, including increases in type 2 cytokines.

**Conclusions:**

Our data indicate that IL-33 contributes to augmented responses to ozone in obese mice. Obesity and ozone also interacted to promote type 2 cytokine production in γδ T cells and ILC2 in the lungs, which may contribute to the observed effects of IL-33.

**Citation:**

Mathews JA, Krishnamoorthy N, Kasahara DI, Cho Y, Wurmbrand AP, Ribeiro L, Smith D, Umetsu D, Levy BD, Shore SA. 2017. IL-33 drives augmented responses to ozone in obese mice. Environ Health Perspect 125:246–253; http://dx.doi.org/10.1289/EHP272

## Introduction

Ozone (O_3_), a common air pollutant, is an asthma trigger. O_3_ causes asthma symptoms, reduces lung function, and causes airway hyperresponsiveness (AHR) (Foster et al. 2000; Gent et al. 2003; Ji et al. 2011). Indeed, emergency department visits and hospital admissions for asthma increase following days of high ambient O_3_ (Gent et al. 2003; Ji et al. 2011). The majority of the U.S. population is either obese or overweight, and obesity is a risk factor for asthma (Dixon et al. 2010). Both overweight and obesity increase O_3_-induced decrements in lung function, especially in subjects with pre-existing AHR (Alexeeff et al. 2007; Bennett et al. 2007). Acute O_3_ exposure also increases pulmonary mechanics in obese but not lean mice and causes greater increases in airway responsiveness in obese than lean mice (Williams et al. 2013). These observations imply a link between body mass and responses to pollutant triggers of asthma. However, the mechanistic basis for obesity-related changes in pulmonary responses to O_3_ is poorly understood.

O_3_ causes injury to pulmonary epithelial cells (Pino et al. 1992), resulting in an inflammatory response that includes increases in bronchoalveolar lavage (BAL) cytokines and chemokines, including TNFα, and neutrophil recruitment to the lungs (Johnston et al. 2008; Lu et al. 2006; Williams et al. 2013). We have reported that genetic deficiency in either TNFα or TNFR2 attenuates obesity-related increases in BAL neutrophils after acute O_3_ exposure, but actually exacerbates O_3_-induced AHR in obese mice (Williams et al. 2013, 2015). Hence, other factors must also contribute to obesity-related elevations in the response to O_3_.

IL-33, an IL-1 family cytokine, may be one of these factors. IL-33 signals via a complex composed of ST2, the primary binding receptor, and a coreceptor, IL-1R AcP, leading to MyD88- and IRAK-dependent MAP kinase and NF-κB activation. A soluble form of ST2 (sST2) containing the extracellular portion of ST2 can also be generated by alternative splicing (Molofsky et al. 2015). IL-33 and ST2 are genetically associated with asthma (Moffatt et al. 2010). IL-33 is abundantly expressed in epithelial cells and is released upon cell stress or necrosis (Cayrol and Girard 2014), as might be expected after O_3_-induced injury. Indeed, lung IL-33 increases upon O_3_ exposure in lean mice (Yang et al. 2016). In addition, exogenous administration of IL-33 to the lungs induces AHR and causes pulmonary neutrophil recruitment in mice (Barlow et al. 2013; Mizutani et al. 2014), events that also occur after O_3_ exposure. Moreover, these effects of IL-33 involve induction of IL-6, CXCR2 utilizing chemokines, such as CXCL1 and CXCL2, and secretion of type 2 cytokines (Barlow et al. 2013; Mizutani et al. 2014). Obesity also augments O_3_-induced increases in BAL CXCL1 and CXCL2, and BAL concentrations of the type 2 cytokines, IL-13 and IL-5 (Johnston et al. 2008; Williams et al. 2013). Hence, we examined the hypothesis that IL-33 contributes to obesity-related increases in the response to O_3._ To do so, we treated lean wildtype (WT) and obese *db/db* mice with an ST2 blocking or isotype antibody prior to O_3_ exposure. Our results indicate that IL-33 contributes to the augmented response to O_3_ in obese mice and that innate lymphoid cells type 2 (ILC2), important targets of IL-33 (Barlow et al. 2013), are activated by O_3_ exposure in obese mice. However, we show that IL-13 producing γδ T cells are also targets of IL-33 and that γδ T cells are required for augmented responses to O_3_ in obese mice. To our knowledge, this is the first report that *pulmonary* γδ T cells express the IL-33 receptor, ST2, and can produce IL-13.

## Methods

### Animals

Female *db/db* mice, which lack the longform of the receptor for the satiety hormone, leptin, and age-matched WT mice (C57BL/6J) were purchased from Jackson Laboratory (Bar Harbor, ME) at 6 weeks old and acclimated in our vivarium for 4 weeks, when the *db/db* mice weighed twice as much as WT mice. Breeding pairs of WT or TCRδ^–/–^ were purchased from Jackson Laboratory and bred in house. After weaning, WT and TCRδ^–/–^ mice were placed on either a high-fat diet (HFD) in which 60% of calories derive from fat (D12451, Research Diets) or normal mouse chow (PicoLab 5053, LabDiet) in which about 13% of calories derive from fat. Mice were maintained on these diets for 24 weeks, at which time HFD-fed mice were obese. There was no difference in body mass in TCRδ^–/–^ and WT mice fed chow (31.6 ± 0.7 versus 32.3 ± 0.7 g, respectively), but HFD-fed TCRδ^–/–^ mice, though still obese, weighed less than HFD-fed WT mice (46.6 ± 0.9 versus 52.1 ± 1.2 g respectively, *p* < 0.01). All protocols were approved by the Harvard Medical Area Standing Committee on Animals. Animals were treated humanely and with regard for alleviation of suffering.

### Protocol

To assess the role of IL-33 in pulmonary responses to O_3_, WT and *db/db* mice were treated with an antibody directed against the extracellular domain of recombinant murine ST2 (10 mg/kg, i.p.) or with isotype (IgG1) antibodies. At this dose, anti-ST2 blocks responses to exogenous IL-33 in mice (Palmer et al. 2009). Mice were exposed to O_3_ 24 hr later, and evaluated 24 hr after exposure. Evaluation included measurement of airway responsiveness, BAL, and lung tissue and blood harvest.

To evaluate the role of CD4 cells in O_3_-induced changes in type 2 cytokines, we depleted CD4 cells. *Db/db* mice were injected once with anti-CD4 (clone: GK1.5, Biolegend) (8 mg/kg) or isotype antibody (Rice and Bucy 1995). Mice were exposed to O_3_ 6 days later and evaluated as described above. Confirmation of CD4 depletion in lung tissue was assessed by flow cytometry.

To examine the role of γδ T cells in obese mice, WT and TCRδ^–/–^ mice were fed either an HFD or normal chow, and then exposed to air or to O_3_, and evaluated as described above.

Methods for BAL, measurement of cytokines and chemokines, RNA extraction and RT-qPCR, and flow cytometry were as previously described (Krishnamoorthy et al. 2015; Williams et al. 2013) and are found in an online supplement (see “Supplemental Methods” in the Supplemental Material).

### Ozone Exposure

Mice were placed in individual wire mesh cages without access to water or food and acutely exposed to air or O_3_ (2 ppm for 3 hr) as described by Williams et al. (2013). Immediately upon cessation of exposure mice were transferred to regular cages with free access to food and water.

### Measurement of Airway Responsiveness

Mice were anesthetized and instrumented for measurement of pulmonary mechanics and airway responsiveness to methacholine, using the forced oscillation technique, as previously described by Williams et al. (2015). A positive end expiratory pressure of 3 cm H_2_O was applied and the chest wall opened to expose the lungs to atmospheric pressure. Changes in total pulmonary resistance (R_L_), Newtonian resistance (Rn), which mainly reflects changes in the mechanical properties of the airways, and the coefficients of lung tissue damping (G) and lung tissue elastance (H), measures of changes in the lung periphery, including airway closure, were assessed after aerosolized saline and after increasing doses of aerosolized methacholine.

### Statistics

Data were analyzed by factorial ANOVA using STATISTICA software (StatSoft®, Tulsa, OK) with mouse genotype, antibody treatment, and exposure or mouse genotype, diet, and exposure as main effects. Fisher’s least significant difference test was used as a post-hoc test. BAL cells and flow cytometry data were log transformed prior to statistical analysis in order to conform to a normal distribution. A *p*-value < 0.05 was considered statistically significant.

## Results

### IL-33 Contributes to Pulmonary Responses to O_3_ in Obese but not Lean Mice

**Figure 1:**
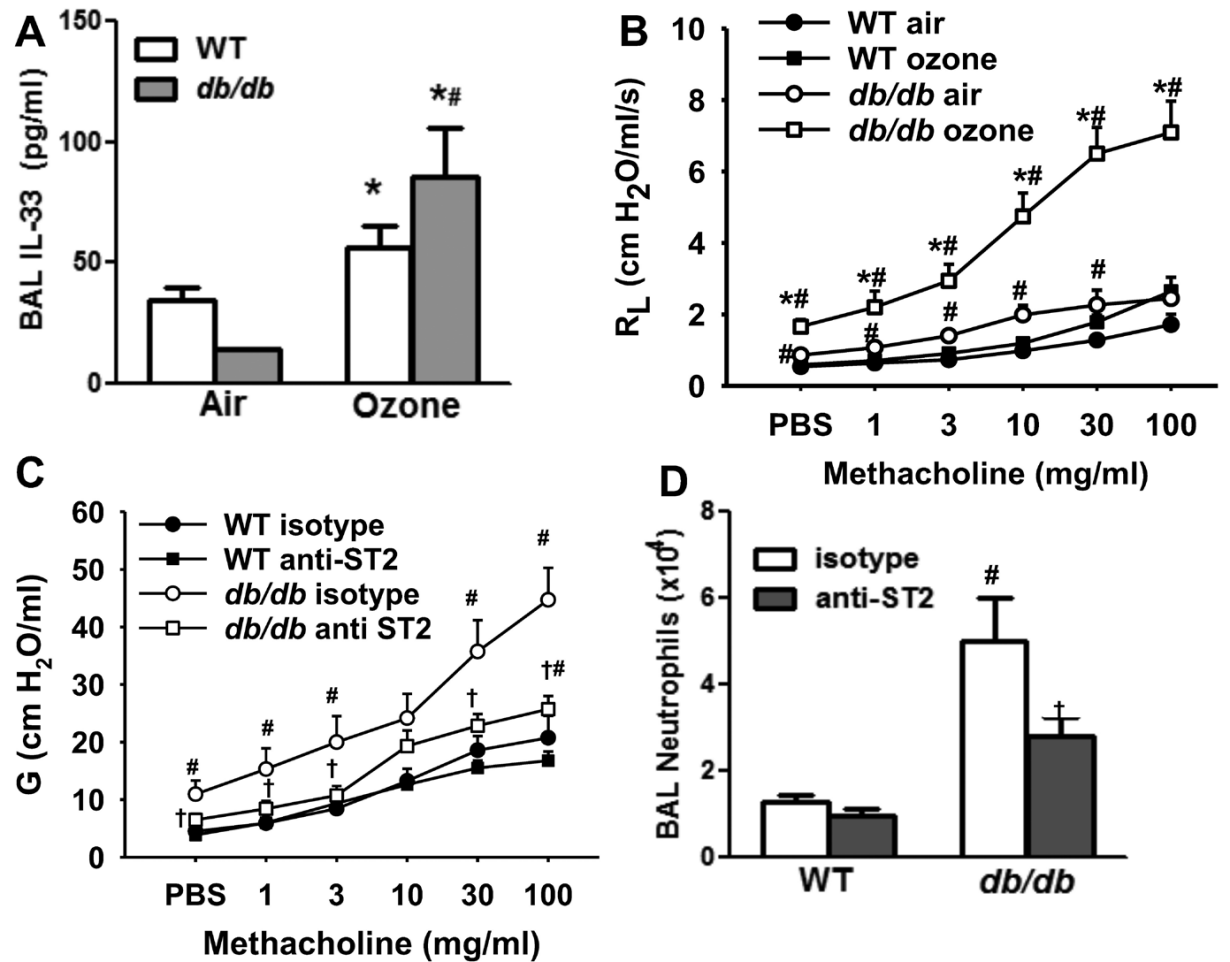
Role of IL-33 in pulmonary responses to O_3_ exposure in obese mice. (*A*) Bronchoalveolar lavage (BAL) IL-33 and (*B*) changes in pulmonary resistance (R_L_) induced by inhaled aerosolized methacholine in lean wildtype (WT) and obese *db/db* female mice exposed to air or ozone (O_3_) (2 ppm for 3 hr) and studied 24 hr after exposure. (*C*) Airway responsiveness to methacholine assessed using G, the coefficient of lung tissue damping, and (*D*) BAL neutrophils in a different cohort of WT and *db/db* treated with isotype or anti-ST2 antibody prior to O_3_ exposure. For panels *A* and *B*, results are mean ± SE of 4–8 mice/group studied over 16 experimental days. For panels *C* and *D*, results are mean ± SE of 4–8 mice/group studied over 8 experimental days. *p < 0.05 versus air. ^#^ *p* < 0.05 versus lean mice with same exposure. ^†^ *p* < 0.05 versus isotype-treated mice of same genotype.

Compared to air, O_3_ exposure increased BAL IL-33, but the effect was significantly greater in obese *db/db* mice than in lean WT mice (Figure 1A). In contrast, serum IL-33 was unchanged by obesity (7.1 ± 0.8 versus 6.0 ± 0.7 pg/mL in O_3_-exposed *db/db* versus WT mice, respectively) and was approximately 50% lower after O_3_ than air in both WT and *db/db* mice (data not shown).

In air exposed mice, baseline pulmonary resistance (R_L_) was greater in *db/db* than WT mice (PBS values in Figure 1B) consistent with the smaller lungs of the *db/db* mice (Lu et al. 2006). O_3_ increased baseline R_L_ in *db/db* but not WT mice (Figure 1B). O_3_ also increased methacholine-induced changes in R_L_ to a greater extent in *db/db* than WT mice (Figure 1B). Essentially similar results were observed for the coefficients of G and for elastance H, measures of the lung periphery and for Rn, a measure of the central airways (see Figure S1A–C). However, the effect was greatest for G, suggesting that the effects of O_3_ are largely mediated in the lung periphery. Consequently, in subsequent analyses of airway responsiveness, methacholine-induced changes in G are presented.

Effects of anti-ST2 treatment were assessed in a separate cohort of WT and *db/db* mice exposed to O_3_. Compared to isotype antibody, anti-ST2 treatment had no effect on airway responsiveness in O_3_-exposed WT mice (Figure 1C). However, in O_3_-exposed *db/db* mice, anti-ST2 treatment significantly reduced baseline G, and significantly reduced airway responsiveness (Figure 1C). Similar results were obtained for R_L_ (see Figure S1D). BAL neutrophils were greater in O_3_-exposed *db/db* versus WT mice (Figure 1D) treated with isotype antibody. Anti-ST2 significantly reduced BAL neutrophils in *db/db* but not in WT mice. Taken together, the results indicate a role for IL-33 in responses to O_3_ in obese mice. In contrast, we observed no significant effect of anti-ST2 versus isotype antibody treatment on airway responsiveness in air-exposed *db/db* mice (see Figure S1E).

### IL-33 Dependent BAL Cytokines and Chemokines

**Figure 2:**
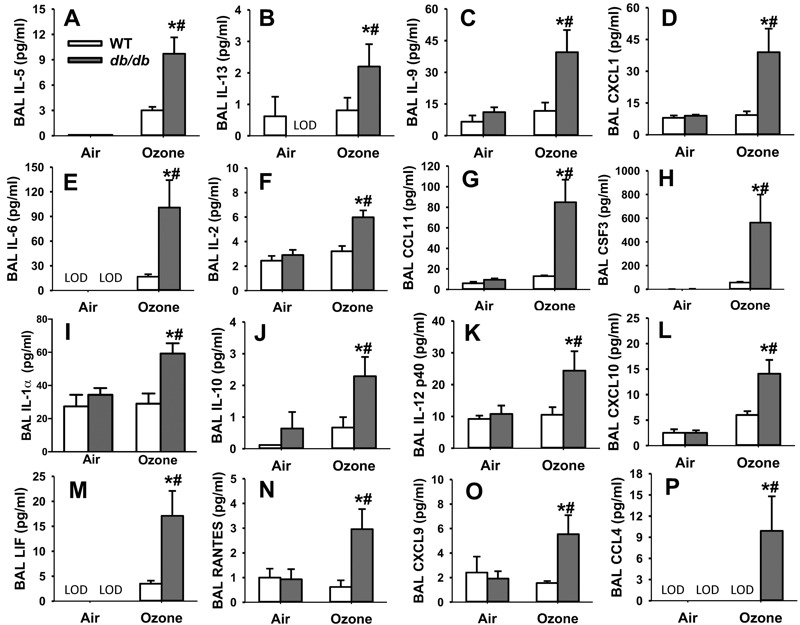
Obesity augments O_3_-induced increases in BAL cytokines, chemokines, and growth factors. BAL (*A*) IL-5, (*B*) IL-13, (*C*) IL-9, (*D*) CXCL1, (*E*) IL-6, (*F*) IL-2, (*G*) eotaxin (CCL11), (*H*) CSF3, (*I*) IL-1α, (*J*) IL-10, (*K*) IL-12 (p40), (*L*) CXCL10, (*M*) LIF, (*N*) RANTES, (*O*) CXCL9, and (*P*) CCL4 in a cohort of WT and *db/db* treated with isotype antibody prior to air or O_3_ exposure. Samples that were undetectable were assigned a value of 0. Limit of detection (LOD) indicates that all samples in the group were below the limit of detection. Note: Results are mean ± SE of 4–7 mice/group studied over 16 experimental days. *p < 0.05 versus air. ^#^ *p* < 0.05 versus lean mice with same exposure.

**Figure 3:**
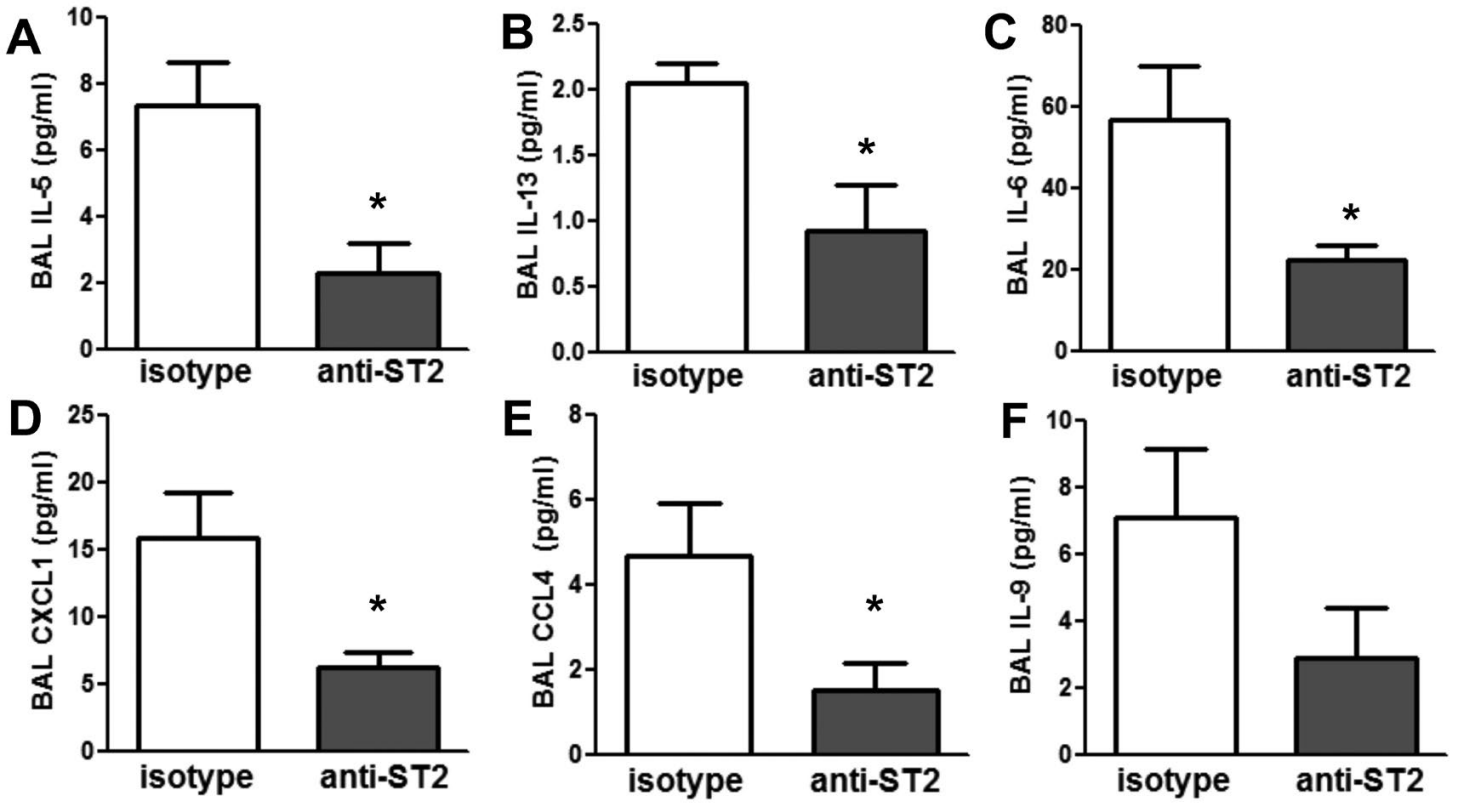
Anti-ST2 reduces BAL cytokines and chemokines in obese O_3_-exposed mice. *Db/db* mice were treated intraperitoneal with ST2-blocking or isotype antibodies 24 hr prior to O_3_ exposure (2 ppm for 3 hr). BAL concentrations of (*A*) IL-5, (*B*) IL-13, (*C*) IL-6, (*D*) CXCL1, (*E*) CCL4, and (*F*) IL-9 were measured by ELISA or by multiplex using BAL that had been concentrated five times. Note: Results are the mean ± SE of 6–10 mice/group studied over 8 experimental days. *p < 0.05 versus isotype treated mice.

Others have reported that exogenously administered IL-33 causes AHR and increases BAL neutrophils by inducing both type 2 cytokines like IL-13 and IL-5, chemokines that utilize CXCR2, like CXCL1 and CXCL2, and IL-6 (Barlow et al. 2013; Chang et al. 2013; Mizutani et al. 2014). O_3_ exposure significantly increased BAL concentrations of the type 2 cytokines IL-5, IL-13, and IL-9 in *db/db* but not WT mice (Figure 2A–C). Similar results were obtained in obese *Cpe^fat^* mice versus their WT controls (Williams et al. 2013). In addition to IL-33, two other epithelial derived cytokines, IL-25 and TSLP, can also induce the secretion of type 2 cytokines. However, neither *Il25* nor *Tslp* expression was affected by O_3_ exposure (data not shown). In addition to IL-5, IL-13 and IL-9, O_3_ also caused greater increases in BAL CXCL1, IL-6, IL-2, eotaxin (CCL11), CSF3, IL-1α, IL-10, IL-12 (p40), CXCL10, LIF, RANTES, CXCL9, and CCL4 in the same cohort of O_3_-exposed isotype-treated *db/db* versus WT mice (Figure 2D–P). Of these, BAL concentrations of IL-5, IL-13, IL-6, CXCL1 and CCL4 were significantly reduced in anti-ST2 versus isotype treated *db/db* mice exposed to O_3_ (Figure 3A–E). A similar effect of anti-ST2 was observed on BAL IL-9, but did not reach statistical significance (Figure 3F). ST2-dependent changes in these cytokines and chemokines (Figure 3) likely contribute to the ST2- dependent effects of O_3_ observed in obese mice (Figure 1C,D).

### Cellular Sources of Type 2 Cytokines

O_3_ causes IL-6 and CXCL family chemokine release from airway epithelial cells and macrophages (Kasahara et al. 2014; McCullough et al. 2014). These cells are the likely targets of ST2-mediated changes in IL-6 and CXCL1 (Figure 3). Indeed, both epithelial cells and macrophages express ST2 and can respond to IL-33 (Cayrol and Girard 2014; Yagami et al. 2010; Yang et al. 2013). Regarding the cellular source of the observed IL-33-dependent type 2 cytokines (Figure 3A,B,F), many cells in the lung, including Th2 cells, macrophages, mast cells, and innate lymphoid cells type 2 (ILC2), have the capacity to release type 2 cytokines after IL-33 stimulation (Chang et al. 2013; Molofsky et al. 2015). γδ T cells also express receptors for IL-33 (Duault et al. 2016) and can produce type 2 cytokines (Inagaki-Ohara et al. 2011), though effects of IL-33 on γδ T cell production of type 2 cytokines have not previously been described. We were unable to detect changes in IL-13^+^ macrophages in obese mice after O_3_ exposure using flow cytometry, nor could we find any evidence of acute mast cell activation within the airways of obese O_3_-exposed mice using ELISA assay of BAL mast cell tryptase (data not shown) suggesting instead a lymphoid source for the observed changes in type 2 cytokines.

IL-13^+^ Th2 cells are elevated in lungs of obese versus lean mice exposed to O_3_ (Williams et al. 2013). To examine the contribution of CD4^+^ cells to the elevations in BAL type 2 cytokines observed in obese O_3_-exposed mice, we depleted these cells with an anti-CD4 antibody. Flow cytometry indicated an approximate 75% reduction in lung CD4^+^ cells, confirming the efficacy of the depletion strategy (see Figure S2A). However, BAL type 2 cytokines were not significantly reduced in O_3_-exposed *db/db* mice treated with anti-CD4 versus isotype antibody (see Figure S2B–D), suggesting that Th2 cells were not the source of the elevated BAL type 2 cytokines observed in these mice (Figure 2), nor were other ST2 dependent cytokines (CXCL1, IL-6, CCL4) affected (see Figure S2E–G). In addition, depletion of CD4 cells had no effect on ozone-induced AHR or BAL neutrophils (see Figure S2G,H).

**Figure 4:**
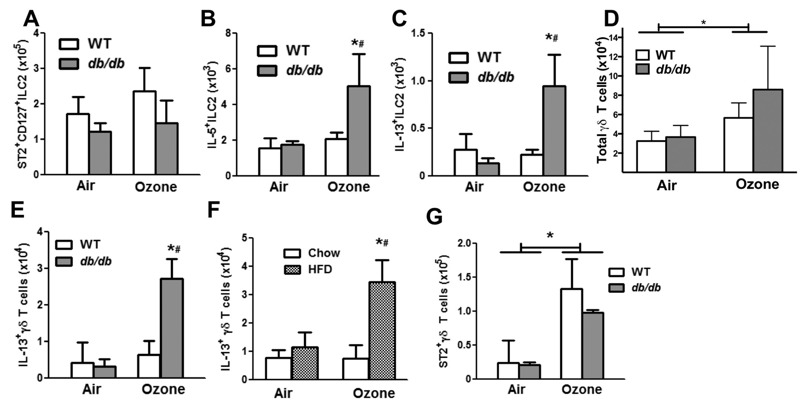
O_3_ increases IL-5^+^ and IL-13^+^ ILC2 cells and IL-13^+^ γδ T cells in obese mice. Flow cytometry was used to assess (*A*) total and (*B* and *C*) activated lung ILC2s in* db/db *and WT mice exposed to air or O_3_. Total ILC2 cells were gated as negative for lineage markers and positive for CD45, ST2, Thy1.2, and CD127. IL-5^+^ and IL-13^+^ ILC2 cells were gated as negative for lineage markers and positive for CD45 and (*B*) IL-5 or (*C*) IL-13 as shown in Figures S3 and S4. Flow cytometry was also used to assess (*D*) total γδ T as gated as positive for CD45, CD3, and TCRδ. (*E*) IL-13+ γδ T cells in air- and O_3_-exposed WT and *db/db* or (*F*) high fat diet (HFD) and chow-fed mice. IL-13^+^ γδ T cells were gated as positive for IL-13, CD45, CD3, and TCRδ. (*G*) ST2^+^ γδ T cells in air- and O_3_-exposed WT and *db/db* mice. ST2^+^ γδ T cells were gated as SSC^low^TCRδ^+^ST2^+^ as shown in Figure S5. Note: Results are the mean ± SE of 4–9 mice/group studied over 3 experimental days. *p < 0.05 versus air. ^#^ *p* < 0.05 versus WT mice with the same exposure.

**Figure 5:**
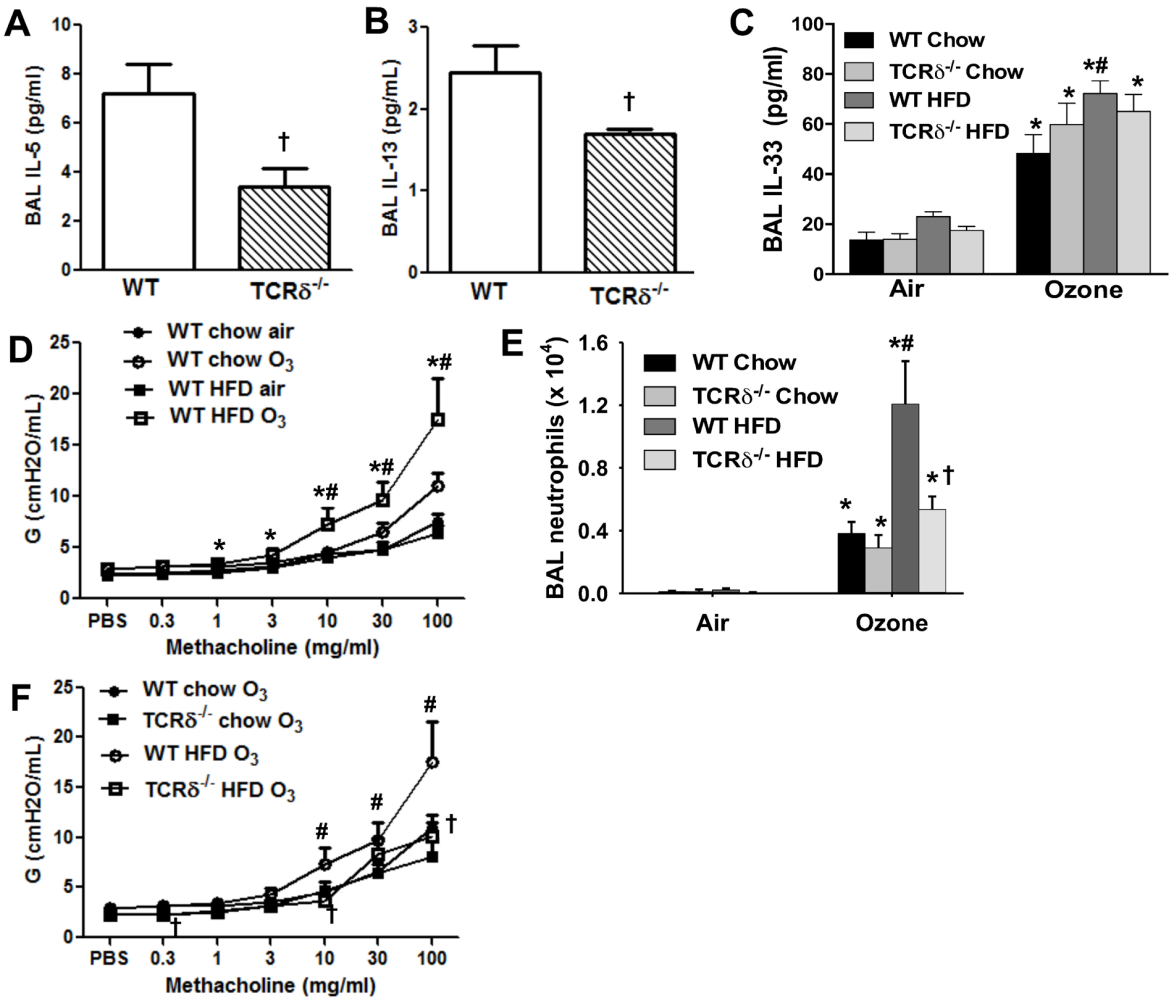
γδ T cells are required for O_3_-induced increases in type 2 cytokines in obese mice. BAL IL-5 (*A*) and IL-13 (*B*) from O_3_-exposed HFD fed WT and TCRδ^–/–^ mice. IL-13 and IL-5 were measured by ELISA after approximately 5× concentration of BAL fluid. (*C*) BAL IL-33 in air and O_3_-exposed chow and HFD fed WT and TCRδ^–/–^ mice. Airway responsiveness, assessed using the coefficient of lung tissue damping–G, and the number of BAL neutrophils in obese (HFD) and lean (chow) WT mice exposed to air or O_3_ (*D*) or WT and TCRδ^–/–^ mice fed a chow or HFD diet and exposed to air or O_3_ (*E*); and airway responsiveness in WT and TCRδ^–/–^ mice fed a chow or HFD diet and exposed to O_3_ (*F*). Note: Results are the mean ± SE of 4–9 mice/group studied over 15 experimental days. *p < 0.05 versus air. ^#^ *p* < 0.05 versus lean mice with same exposure. ^†^ *p* < 0.05 WT versus TCRδ^–/–^ mice with same diet and exposure.

Instead, ILC2 and/or γδ T cells appear to account for IL-33 dependent changes in BAL type 2 cytokines observed in obese O_3_-exposed mice. Flow cytometry indicated no change with obesity or O_3_ in the total number of pulmonary ILC2s (Figure 4A). However, there was an increase in cytokine production by ILC2: O_3_ increased the number of IL-5^+^ and IL-13^+^ ILC2s in obese *db/db* but not lean wildtype mice (Figure 4B,C; see also Figures S3 and S4), consistent with the observed changes in BAL IL-5 and IL-13 (Figure 2A,B). O_3_ also increased the number of pulmonary IL-13^+^ γδ T cells in *db/db* but not WT mice (Figure 4E). Total pulmonary γδ T cells were not affected by obesity but were increased by O_3_ in both *db/db* and WT mice (Figure 4D). O_3_ also increased IL-13^+^ γδ T cells in mice with dietary obesity but not in lean controls (Figure 4F). IL-5^+^ γδ T cells were not assessed. Importantly, after O_3_ exposure, BAL IL-5 and IL-13 were lower in obese TCRδ^–/–^ mice, which lack γδ T cells, than in obese WT mice (Figure 5A,B), indicating that γδ T cells likely contributed to increases in these cytokines observed in obese O_3_ exposed mice (Figure 2). Because type 2 cytokines were also dependent on ST2 (Figure 3), we determined whether γδ T cells can respond to IL-33. Importantly, some pulmonary γδ T cells expressed the ST2 receptor (see Figure S5) and the number of ST2^+^ γδ T cells was greater in O_3_ than in air exposed mice (Figure 4G). To confirm that these ST2^+^ γδ T cells could produce IL-13, we co-stained lung cells with antibodies to both ST2 and IL-13. In these experiments, only O_3_-exposed *db/db* mice were used, since we observed little or no IL-13 in air exposed mice or in WT mice exposed to O_3_ (Figure 2B). Our data indicate that approximately 5 ± 1.5% (*n* = 3) of the γδ T cells in O_3_-exposed *db/db* mice were IL-13^+^. Importantly, virtually all (> 85%) of these IL-13^+^ γδ T were also ST2^+^ (see Figure S5).

Whereas γδ T cell deficiency reduced BAL type 2 cytokines, it did not affect BAL CXCL1 or IL-6 in obese O_3_ exposed mice (data not shown), indicating that other IL-33 target cells, perhaps epithelial cells or macrophages, are the source of these ST2-dependent cytokines. The reduction in BAL IL-5 and BAL IL-13 in obese TCRδ^–/–^ versus obese WT mice exposed to O_3_ (Figure 5A,B) was not because of differences in the ability of TCRδ^–/–^ versus WT mice to generate IL-33: BAL IL-33 was not lower in obese TCRδ^–/–^ versus obese WT mice exposed to O_3_ (Figure 5C), although O_3_ caused greater increases in BAL IL-33 in obese HFD versus lean chow fed mice (Figure 5C), as it did in *db/db* versus WT mice (Figure 1A).

O_3_ caused greater increases in airway responsiveness (Figure 5D) and greater increases in BAL neutrophils (Figure 5E) in HFD than chow fed mice, similar to the results obtained in genetically obese mice (Figure 1). Importantly, after O_3_, both airway responsiveness (Figure 5F) and BAL neutrophils (Figure 5E) were lower in obese TCRδ^–/–^ than obese WT mice. Taken together, our data indicate that γδ T cells are required for the augmented responses to O_3_ observed in obese mice, perhaps as a consequence of their ability to produce type 2 cytokines in response to IL-33. Of note, although HFD fed TCRδ^–/–^ mice weighed somewhat less than HFD fed WT mice, multiplex analysis indicated no significant difference in serum cytokines and chemokines in these two groups of mice after air exposure except for an *increase* in serum IL-1α in the HFD fed TCRδ^–/–^ mice (data not shown). The data suggest that the difference in body mass in the two groups of HFD fed mice may did not appear to be biologically significant.

## Discussion

Our data indicate that the augmented responses to O_3_ observed in obese mice are partially dependent on IL-33 (Figure 1). IL-6, CXCL1, and type 2 cytokines likely contributed to the effects of IL-33 (Figures 2 and 3), and we identified ILC2s and γδ T cells as sources of IL-33 dependent type 2 cytokines in obese O_3_-exposed mice (Figures 4 and 5). Finally, we demonstrated that γδ T cell deficiency reduced obesity-related increases in the response to O_3_, and reduced associated type 2 cytokine production (Figure 5).

IL-33 contributed to obesity-related increases in the response to O_3_: BAL IL-33 was greater in obese than lean O_3_ exposed mice (Figures 1A and 5C) and anti-ST2 reduced O_3_-induced increases in baseline mechanics, in airway responsiveness, and in BAL neutrophils in obese but not lean mice (Figure 1C,D). Effects of IL-33 on the neutrophil chemotactic factors, CXCL1 and IL-6, are likely involved in the changes in BAL neutrophils (Figure 1D): both CXCL1 and IL-6 were elevated in O_3_-exposed obese versus lean mice (Figure 2D,E) and reduced in these mice by anti-ST2 treatment (Figure 3), and both IL-6 and CXCL1 are required for O_3_-induced increases in BAL neutrophils, including in obese mice (Johnston et al. 2005; Lang et al. 2008). Furthermore, exogenous IL-33 induces IL-6 and CXCL1 expression in the lungs (Mizutani et al. 2014). However, reductions in IL-13 by anti-ST2 (Figure 3A) may have also contributed to the anti-ST2-dependent reduction in BAL neutrophils (Figure 1D), since anti-IL-13 also reduces BAL neutrophils in obese O_3_-exposed mice (Williams et al. 2013). A role for IL-13 would also explain the efficacy of anti-ST2 in *db/db* but not wildtype mice, since O_3_ increased BAL IL-13 only in the obese mice (Figure 2), consistent with previous observations in obese *Cpe^fat^* mice (Williams et al. 2013).

Anti-ST2 also attenuated O_3_-induced increases in baseline pulmonary mechanics in *db/db* mice (Figure 1C; see also Figure S1D). A similar reduction is observed after anti-IL-13 in obese mice (Williams et al. 2013), suggesting that IL-33-dependent increases in IL-13 contributed to obesity-related increases in effects of O_3_ on baseline pulmonary mechanics (Figure 1B, PBS values). However, blocking IL-13 does not reduce AHR in O_3_-exposed obese mice (Williams et al. 2013), whereas blocking ST2 did (Figure 1C). Thus, other IL-33-driven factors must also have a role. IL-9 may be one of these factors. IL-9 is ST2-dependent (Gerlach et al. 2014), was increased by O_3_ in obese but not lean mice (Figure 2C), and can induce AHR (Goswami and Kaplan 2011). Chemokines such as CXCL1, which was reduced in anti-ST2 versus isotype treated obese mice (Figure 3D), may also contribute to the observed ST2-dependent effects on AHR in obese mice: blocking CXCR2 reduces AHR induced by exogenous IL-33 (Mizutani et al. 2014), and CXCR2 is required for O_3_-induced AHR in lean mice (Johnston et al. 2005).

BAL IL-5 and IL-13 were reduced in anti-ST2 versus isotype antibody treated obese mice exposed to O_3_ and a similar trend was observed for IL-9 (Figure 3), indicating a requirement for IL-33 in the induction of these type 2 cytokines by O_3_. Of note, although we did find increased type 2 cytokines we could not find eosinophils in the BAL or lung tissue of either obese or lean mice after O_3_. Our data provided little evidence that macrophages, mast cells, or CD4^+^ T cells were the IL-33 target cells involved in production of type 2 cytokines in obese mice after O_3_, although we cannot rule them out entirely. Indeed, because *db/db* mice have thymic atrophy (Palmer et al. 2006), it is possible that in other types of obese mice, CD4^+^ cells do play a role in the type 2 cytokine production observed following O_3_. However, in *db/db* mice, ILC2s and γδ T cells were the likely sources of these cytokines. O_3_ increased IL-5^+^ and IL-13^+^ ILC2 cells in lungs of obese but not lean mice after O_3_ (Figure 4B,C; see also Figure S4) without changes in the total number of ILC2s (Figure 4A; see also Figure S3). A recent report indicates that O_3_ can also induce type 2 cytokine production from ILC2s from lungs of lean BALB/c mice (Yang et al. 2016). Importantly, the authors also noted that lean BALB/c mice had substantive increases in BAL IL-5 release after O_3_, whereas lean C57BL/6 mice had only minimal changes in BAL IL-5, consistent with our observations with the latter strain (Figure 2A). Importantly, reconstitution of lung ILC2s into mice lacking these cells restores their ability to develop AHR after O_3_ exposure, indicating that activation of ILC2 by O_3_ does indeed have the capacity to cause AHR (Yang et al. 2016). ILC2 also appear to be the source of type 2 cytokines induced by O_3_ exposure in the nose (Kumagai et al. 2016). Taken together, the data extend the list of asthma triggers that can induce ILC2 activation to include not only allergy and viral infection (Chang et al. 2013; Vercelli et al. 2014), but also O_3_, and IL-33 seems to be a common denominator inducing their activation in each instance.

We also observed increased IL-13^+^ γδ T cells in obese but not lean mice after O_3_ exposure (Figure 4E,F). Importantly, these cells also expressed ST2 (see Figure S5). γδ T cells produce type 2 cytokines in other tissues (Inagaki-Ohara et al. 2011; Qi et al. 2009) and undergo proliferation in response to IL-33 (Duault et al. 2016). However, to our knowledge, these data are the first to show that pulmonary γδ T cells can produce IL-13 (Figure 4E,F) and that IL-33 can induce IL-13 expression in γδ T cells. BAL IL-5 and IL-13 were reduced in obese TCRδ^–/–^ versus WT mice after O_3_ exposure (Figure 5A,B). These reductions could be the result of factors produced from γδ T cells acting to promote type 2 cytokine expression in ILC2s. However, given our observations that γδ T cells expressed ST2 receptors, especially after O_3_ (Figure 5F; see also Figure S5), and that IL-13^+^ γδ T cells were also ST2^+^ (see Figure S5), our data are consistent with the hypothesis that γδ T cells are themselves a source of ST2-dependent IL-13 and IL-5 in obese O_3_-exposed mice. Follow up experiments will be required to determine the relative roles of ILC2 versus γδ T cells in these events. Others have also reported a role for γδ T cells in O_3_-induced AHR in lean mice (King et al. 1999; Matsubara et al. 2009). The ability of γδ T cells to express ST2 receptors and produce type 2 cytokines now needs to be considered in experimental interventions designed to identify the cellular locus of action of IL-33.

Recent reports by others indicate profound systemic effects of O_3_ exposure that include elevations in circulating glucose and lipids (Miller et al. 2015). Hence, we cannot rule out the possibility that the observed effects of γδ T cell deficiency (Figure 5) and anti-ST2 (Figures 1 and 3) were the result of systemic rather than pulmonary effects of O_3_ in obese mice. However, as IL-33 was reduced in the blood, but increased in BAL after O_3_ exposure, the observed effects of IL-33 were more likely the result of IL-33 released in the lung.

In summary, IL-33 contributed to the augmented responses to O_3_ observed in obese mice. Obesity and O_3_ also interacted to induce type 2 cytokine expression in ILC2s and γδ T cells, and these cells appear to contribute to the effects of IL-33, though other cellular targets of IL-33 may also be involved. There was little or no role for IL-33 in lean mice. Thus, our results also highlight obesity-related differences in the regulation of responses to O_3_, and emphasize the need for greater understanding of the effects of O_3_ in obese subjects, who now make up a substantial proportion of the U.S. population.

## Supplemental Material

(576 KB) PDF

## References

[r1] Alexeeff SE, Litonjua AA, Suh H, Sparrow D, Vokonas PS, Schwartz J (2007). Ozone exposure and lung function: effect modified by obesity and airways hyperresponsiveness in the VA Normative Aging Study.. Chest.

[r2] Barlow JL, Peel S, Fox J, Panova V, Hardman CS, Camelo A (2013). IL-33 is more potent than IL-25 in provoking IL-13-producing nuocytes (type 2 innate lymphoid cells) and airway contraction.. J Allergy Clin Immunol.

[r3] Bennett WD, Hazucha MJ, Folinsbee LJ, Bromberg PA, Kissling GE, London SJ (2007). Acute pulmonary function response to ozone in young adults as a function of body mass index.. Inhal Toxicol.

[r4] Cayrol C, Girard JP (2014). IL-33: an alarmin cytokine with crucial roles in innate immunity, inflammation and allergy.. Curr Opin Immunol.

[r5] Chang YJ, DeKruyff RH, Umetsu DT (2013). The role of type 2 innate lymphoid cells in asthma.. J Leukoc Biol.

[r6] Dixon AE, Holguin F, Sood A, Salome CM, Pratley RE, Beuther DA (2010). An official American Thoracic Society Workshop report: obesity and asthma.. Proc Am Thorac Soc.

[r7] Duault C, Franchini DM, Familliades J, Cayrol C, Roga S, Girard JP (2016). TCRVγ9 γδ T cell response to IL-33: a CD4 T cell-dependent mechanism.. J Immunol.

[r8] Foster WM, Brown RH, Macri K, Mitchell CS (2000). Bronchial reactivity of healthy subjects: 18–20 h postexposure to ozone.. J Appl Physiol (1985).

[r9] Gent JF, Triche EW, Holford TR, Belanger K, Bracken MB, Beckett WS (2003). Association of low-level ozone and fine particles with respiratory symptoms in children with asthma.. JAMA.

[r10] Gerlach K, Hwang Y, Nikolaev A, Atreya R, Dornhoff H, Steiner S (2014). TH9 cells that express the transcription factor PU.1 drive T cell-mediated colitis via IL-9 receptor signaling in intestinal epithelial cells.. Nat Immunol.

[r11] Goswami R, Kaplan MH (2011). A brief history of IL-9.. J Immunol.

[r12] Inagaki-Ohara K, Sakamoto Y, Dohi T, Smith AL (2011). γδ T cells play a protective role during infection with *Nippostrongylus brasiliensis* by promoting goblet cell function in the small intestine.. Immunology.

[r13] Ji M Cohan DS Bell ML 2011 Meta-analysis of the association between short-term exposure to ambient ozone and respiratory hospital admissions. Environ Res Lett 6 2 024006, doi:10.1088/1748-9326/6/2/024006 21779304 PMC3138529

[r14] Johnston RA, Mizgerd JP, Shore SA (2005). CXCR2 is essential for maximal neutrophil recruitment and methacholine responsiveness after ozone exposure.. Am J Physiol Lung Cell Mol Physiol.

[r15] Johnston RA, Theman TA, Lu FL, Terry RD, Williams ES, Shore SA (2008). Diet-induced obesity causes innate airway hyperresponsiveness to methacholine and enhances ozone-induced pulmonary inflammation.. J Appl Physiol (1985).

[r16] Kasahara DI, Kim HY, Mathews JA, Verbout NG, Williams AS, Wurmbrand AP (2014). Pivotal role of IL-6 in the hyperinflammatory responses to subacute ozone in adiponectin-deficient mice.. Am J Physiol Lung Cell Mol Physiol.

[r17] King DP, Hyde DM, Jackson KA, Novosad DM, Ellis TN, Putney L (1999). Cutting edge: protective response to pulmonary injury requires γδ T lymphocytes.. J Immunol.

[r18] Krishnamoorthy N, Burkett PR, Dalli J, Abdulnour RE, Colas R, Ramon S (2015). Cutting edge: maresin-1 engages regulatory T cells to limit type 2 innate lymphoid cell activation and promote resolution of lung inflammation.. J Immunol.

[r19] Kumagai K, Lewandowski R, Jackson-Humbles DN, Li N, Van Dyken SJ, Wagner JG (2016). Ozone-induced nasal type 2 immunity in mice is dependent on innate lymphoid cells.. Am J Respir Cell Mol Biol.

[r20] Lang JE, Williams ES, Mizgerd JP, Shore SA (2008). Effect of obesity on pulmonary inflammation induced by acute ozone exposure: role of interleukin-6.. Am J Physiol Lung Cell Mol Physiol.

[r21] Lu FL, Johnston RA, Flynt L, Theman TA, Terry RD, Schwartzman IN (2006). Increased pulmonary responses to acute ozone exposure in obese *db/db* mice.. Am J Physiol Lung Cell Mol Physiol.

[r22] Matsubara S, Takeda K, Jin N, Okamoto M, Matsuda H, Shiraishi Y (2009). Vγ1+ T cells and tumor necrosis factor-alpha in ozone-induced airway hyperresponsiveness.. Am J Respir Cell Mol Biol.

[r23] McCullough SD, Duncan KE, Swanton SM, Dailey LA, Diaz-Sanchez D, Devlin RB (2014). Ozone induces a proinflammatory response in primary human bronchial epithelial cells through mitogen-activated protein kinase activation without nuclear factor-κB activation.. Am J Respir Cell Mol Biol.

[r24] Miller DB, Karoly ED, Jones JC, Ward WO, Vallanat BD, Andrews DL (2015). Inhaled ozone (O_3_)-induces changes in serum metabolomic and liver transcriptomic profiles in rats.. Toxicol Appl Pharmacol.

[r25] Mizutani N, Nabe T, Yoshino S (2014). IL-17A Promotes the exacerbation of IL-33-induced airway hyperresponsiveness by enhancing neutrophilic inflammation via CXCR2 signaling in mice.. J Immunol.

[r26] Moffatt MF, Gut IG, Demenais F, Strachan DP, Bouzigon E, Heath S (2010). A large-scale, consortium-based genomewide association study of asthma.. N Engl J Med.

[r27] Molofsky AB, Savage AK, Locksley RM (2015). Interleukin-33 in tissue homeostasis, injury, and inflammation.. Immunity.

[r28] Palmer G, Aurrand-Lions M, Contassot E, Talabot-Ayer D, Ducrest-Gay D, Vesin C (2006). Indirect effects of leptin receptor deficiency on lymphocyte populations and immune response in *db/db* mice.. J Immunol.

[r29] Palmer G, Talabot-Ayer D, Lamacchia C, Toy D, Seemayer CA, Viatte S (2009). Inhibition of interleukin-33 signaling attenuates the severity of experimental arthritis.. Arthritis Rheum.

[r30] Pino MV, Levin JR, Stovall MY, Hyde DM (1992). Pulmonary inflammation and epithelial injury in response to acute ozone exposure in the rat.. Toxicol Appl Pharmacol.

[r31] Qi Q, Xia M, Hu J, Hicks E, Iyer A, Xiong N (2009). Enhanced development of CD4^+^ γδ T cells in the absence of Itk results in elevated IgE production.. Blood.

[r32] Rice JC, Bucy RP (1995). Differences in the degree of depletion, rate of recovery, and the preferential elimination of naive CD4+ T cells by anti-CD4 monoclonal antibody (GK1.5) in young and aged mice.. J Immunol.

[r33] Vercelli D, Gozdz J, von Mutius E (2014). Innate lymphoid cells in asthma: when innate immunity comes in a Th2 flavor.. Curr Opin Allergy Clin Immunol.

[r34] Williams AS Mathews JA Kasahara DI Chen L Wurmbrand AP Si H 2013 Augmented pulmonary responses to acute ozone exposure in obese mice: roles of TNFR2 and IL-13. Environ Health Perspect 121 5 551 557, doi:10.1289/ehp.1205880 23434795 PMC3673194

[r35] Williams AS, Mathews JA, Kasahara DI, Wurmbrand AP, Chen L, Shore SA (2015). Innate and ozone-induced airway hyperresponsiveness in obese mice: role of TNF-α.. Am J Physiol Lung Cell Mol Physiol.

[r36] Yagami A, Orihara K, Morita H, Futamura K, Hashimoto N, Matsumoto K (2010). IL-33 mediates inflammatory responses in human lung tissue cells.. J Immunol.

[r37] Yang Q, Ge MQ, Kokalari B, Redai IG, Wang X, Kemeny DM (2016). Group 2 innate lymphoid cells mediate ozone-induced airway inflammation and hyperresponsiveness in mice.. J Allergy Clin Immunol.

[r38] Yang Z Grinchuk V Urban JF Jr Bohl J Sun R Notari L 2013 Macrophages as IL-25/IL-33-responsive cells play an important role in the induction of type 2 immunity. PLoS One 8 3 e59441, doi:10.1371/journal.pone.0059441 23536877 PMC3607614

