# Supplementary material for: IL-33 Drives Augmented Responses to Ozone in Obese Mice
Source: Environ Health Perspect. 2016 Jul 29;125(2):246–53. doi: 10.1289/EHP272 (PMC5289908; doi:10.1289/EHP272)
Supplement: (576 KB) PDF [file EHP272.s001.acco.pdf]

Environ Health Perspect
DOI: 10.1289/EHP272

Note to readers with disabilities: *EHP* strives to ensure that all journal content is accessible to all readers. However, some figures and Supplemental Material published in *EHP* articles may not conform to 508 standards due to the complexity of the information being presented. If you need assistance accessing journal content, please contact ehp508@niehs.nih.gov. Our staff will work with you to assess and meet your accessibility needs within 3 working days.

Supplemental Material

IL-33 Drives Augmented Responses to Ozone in Obese Mice

Joel Mathews, Nandini Krishnamoorthy, David Itiro Kasahara, Youngji Cho, Allison Patricia Wurmbrand, Luiza Ribeiro, Dirk Smith, Dale Umetsu, Bruce D. Levy, and Stephanie Ann Shore

Table of Contents

Supplemental Methods

References

Figure S1. O_3-induced AHR is augmented in obese mice and reduced by anti-ST2. Changes in in the coefficients of A) lung tissue damping (G) and B) elastance (H) and in C) Newtonian resistance (R_n) induced by inhaled aerosolized methacholine in lean wildtype (WT) and obese *db/db* female mice exposed to air or ozone (O_3) (2 ppm for 3 h) and studied 24 h after exposure. D) Changes in pulmonary resistance (R_L) in *db/db* and WT mice treated with isotype or anti-ST2 antibody prior to O_3 exposure. E) Methacholine-induced changes in G in *db/db* mice treated with either anti-ST2 or isotype antibody and exposed to air. Results are mean $\pm$ SE of 4-8 mice/group.

Figure S2. Effect of anti-CD4 on BAL cytokines in obese-O_3 exposed mice. Changes in total lung CD4+ cells (A), and in BAL IL-5 (B), IL-9 (C), KC (D), IL-6 (E), CCL4 (F), Airway hyperresponsiveness (G) and BAL neutrophils (H) in *db/db* mice treated with anti-CD4 or isotype antibody prior to O_3 exposure. Results are mean $\pm$ SE of 4-6 mice/group studied over 4 experimental days.

Figure S3. Gating of ILC2. Lungs were disrupted, and single cell suspensions generated. Unstimulated cells were stained with CD45, Thy1.2, a linage cocktail, ST2, and CD127. In A-D are shown stains from one representative *db/db* mouse exposed to O_3. The fluorescence minus one (FMO) control stains for Lin, Thy1.2, ST2, and CD127 are also shown. For control staining, cells from three *db/db* O_3-exposed mice were combined. Total ILC2 (Figure 4A) were gated as $SSC^{Low}FSC^{Low}CD45^{+}Lin^{-}Thy1.2^{+}ST2^{+}CD127^{+}$.

Figure S4. Gating for IL-13^{+} and IL-5^{+} ILC2. Lungs were disrupted, single cell suspensions generated, and cells stimulated with PMA and ionomycin as described in Supplemental Methods. Shown is a representative stain from one *db/db* O_3-exposed mouse. Cells were stained with antibodies to CD45, Thy1.2, a linage (Lin) cocktail, IL-5 or IL-13, and ST2. IL-13^{+} and IL-5^{+} ILC2s were gated as $SSC^{Low}FSC^{Low}CD45^{+}Lin^{-}Thy1.2^{+}IL\text{-}13^{+}$ and $SSC^{Low}FSC^{Low}CD45^{+}Lin^{-}Thy1.2^{+}IL\text{-}5^{+}$ respectively. As confirmation of the cells ILC2 state, cells were also stained with ST2, shown in the upper right quadrant are those cells that are $SSC^{Low}FSC^{Low}CD45^{+}Lin^{-}Thy1.2^{+}IL\text{-}13^{+}ST2^{High}$ or $SSC^{Low}FSC^{Low}CD45^{+}Lin^{-}Thy1.2^{+}IL\text{-}5^{+}ST2^{High}$ which the majority were. The fluorescence minus one (FMO) control stains for IL-13 and IL-5 (which used the same isotype) are also shown. For control staining, from three *db/db* O_3-exposed mice were combined.

Figure S5. IL-13^{+}ST2^{+} costaining on γδ T cells. Lungs were disrupted, single cell suspensions generated, and cells stimulated with PMA and ionomycin as described in Supplemental Methods. Cells were stained with antibodies to CD45, CD3, TCRδ, IL-13, and ST2. IL-13^{+} γδ T cells (Figure 4C,E) were gated as $SSC^{low}CD45^{+}CD3^{+}TCR\delta^{+}IL\text{-}13^{+}$ and $ST2^{+}$γδ T cells were gated as $SSC^{low}TCR\delta^{+}ST2^{+}$ (Figure 4C,D). To determine if the IL-13^{+} γδ T cells were also positive for ST2, cells were gated as $SSC^{low}CD45^{+}CD3^{+}TCR\delta^{+}IL\text{-}13^{+}ST2^{+}$. Shown is one representative stain from a *db/db* mouse exposed to O_3. For the fluorescence minus one (FMO) control stains, cells from three *db/db* O_3-exposed mice were combined.

Supplemental methods

Protocol: Three cohorts of mice were used for data in Figures 1-3. Figures 1A, 1B, and Figure 2 derived from a cohort of WT and *db/db* mice treated with an isotype antibody and exposed to either air or O_3. Figures 1C and 1D, and Figure 3 were derived from data from another cohort of WT and *db/db* mice treated with either anti-ST2 or isotype antibody and exposed to O_3. Figure S1E was derived from a third cohort of *db/db* mice exposed to air and treated with either isotype or anti-ST2.

Bronchoalveolar lavage: The lungs were lavaged twice with 1 ml cold PBS. The lavagates were pooled and centrifuged and total BAL cells and differentials assessed. BAL supernatants were stored at -80°C until assayed for IL-13 and IL-33 by ELISA (R&D Systems and eBioscience respectively). Other cytokines and chemokines were measured with a multiplex assay (Eve Technologies). To measure BAL cytokines in the anti-ST2, anti-CD4, and HFD experiments, BAL supernatants were first concentrated approximately 5X using Amicon Ultra-0.5 centrifugal filters (3 K device) (Millipore) and then assayed by ELISA or multiplex assay. Concentration of the BAL samples permitted assay of some cytokines/chemokines that were close to the limit of detection of the assay.

RNA extraction and real time PCR: After BAL, a large hole was created in the left ventricle of the heart and the lungs were flushed of blood by injecting 10 ml of cold PBS through the right ventricle. The lungs were then excised and the right lung immersed in RNAlater (Qiagen) for subsequent preparation of RNA as previously described (Shore et al. 2011). RNA concentration and purity was determined using a small volume spectrophotometer (Nanodrop, Thermo Scientific). RNA was converted into cDNA using a commercial kit (SuperScript III for qRT-

PCR, Invitrogen). *Il25* mRNA abundance was quantified using real time PCR (7300 Real-Time PCR Systems, Applied Biosystems) with SYBR-green detection and normalized to *36B4* ribosomal RNA (*Rplp0)*. Primers for *Il25* were forward – TGC TCC AGT CAG CCT CTC TC and reverse – CAC GAT CAT TGC CAA GAA TG). Primers for *Rplp0* were previously described (Shore et al. 2011). Changes in mRNA were computed using the ΔΔCt method.

Flow cytometry: After the blood was flushed from the lungs (see above), the left lung was harvested, chilled in RPMI 1640 media, digested, stimulated (PMA/Ionomycin) and prepared for flow cytometry as previously described (Mathews et al. 2014). For confirmation of CD4 depletion unstimulated spleen and lungs cells were stained using the following antibodies: Alexa Fluor 488 anti-CD4 (clone: GK1.5) and PE-cy7 anti-TCRβ (clone: H57-597). Others have demonstrated that use of GK1.5 to deplete CD4 cells does not interfere with subsequent detection of CD4 using the GK1.5 antibody (De Silva et al. 1998). For ILC staining, we did not perform BAL or measurements of airway responsiveness. Lung cells were harvested and stimulated with PMA and ionomycin as described above. Cells were stained with conjugated antibodies as previously described (Krishnamoorthy et al. 2015) including FITC – anti-mouse lineage cocktail (Biolegend), PE anti-ST2 (Biolegend, clone: DI9), APC anti-IL-13 (eBioscience clone: eBio13A), PE-cy7 anti-Thy1.2 (eBioscience Clone: 53-2.1), and APC anti-CD127 (eBioscience Clone: eBioRDR5). Finally for γδ TCR staining, the following antibodies were used, APC-Strep (Biolegend), or Biotin anti-TCRδ (Biolegend clone: Gl3) or PE anti-TCRδ clone: GL3), PE-cy7 anti-CD45 (clone: 30-F11), Alexa Fluor 647 anti-IL-13 (eBioscience clone: eBio13A) and APC-cy7 anti-CD3 (clone: 17A2), PE anti-ST2 (all antibodies from Biolegend unless noted). Cells were then passed through a flow cytometer (Canto II, BD Biosciences) and data were analyzed using Flowjo software (Tree Star).

References

De Silva HD, Van Driel IR, La Gruta N, Toh BH, Gleeson PA. 1998. CD4+ T cells, but not CD8+ T cells, are required for the development of experimental autoimmune gastritis. Immunology 93(3): 405-408.

Krishnamoorthy N, Burkett PR, Dalli J, Abdulnour RE, Colas R, Ramon S, et al. 2015. Cutting edge: maresin-1 engages regulatory T cells to limit type 2 innate lymphoid cell activation and promote resolution of lung inflammation. J Immunol 194(3): 863-867.

Mathews JA, Williams AS, Brand JD, Wurmbrand AP, Chen L, Ninin FM, et al. 2014. gammadelta T cells are required for pulmonary IL-17A expression after ozone exposure in mice: role of TNFalpha. PLoS One 9(5): e97707.

Shore SA, Williams ES, Chen L, Benedito LA, Kasahara DI, Zhu M. 2011. Impact of aging on pulmonary responses to acute ozone exposure in mice: role of TNFR1. Inhal Toxicol 23(14): 878-888.

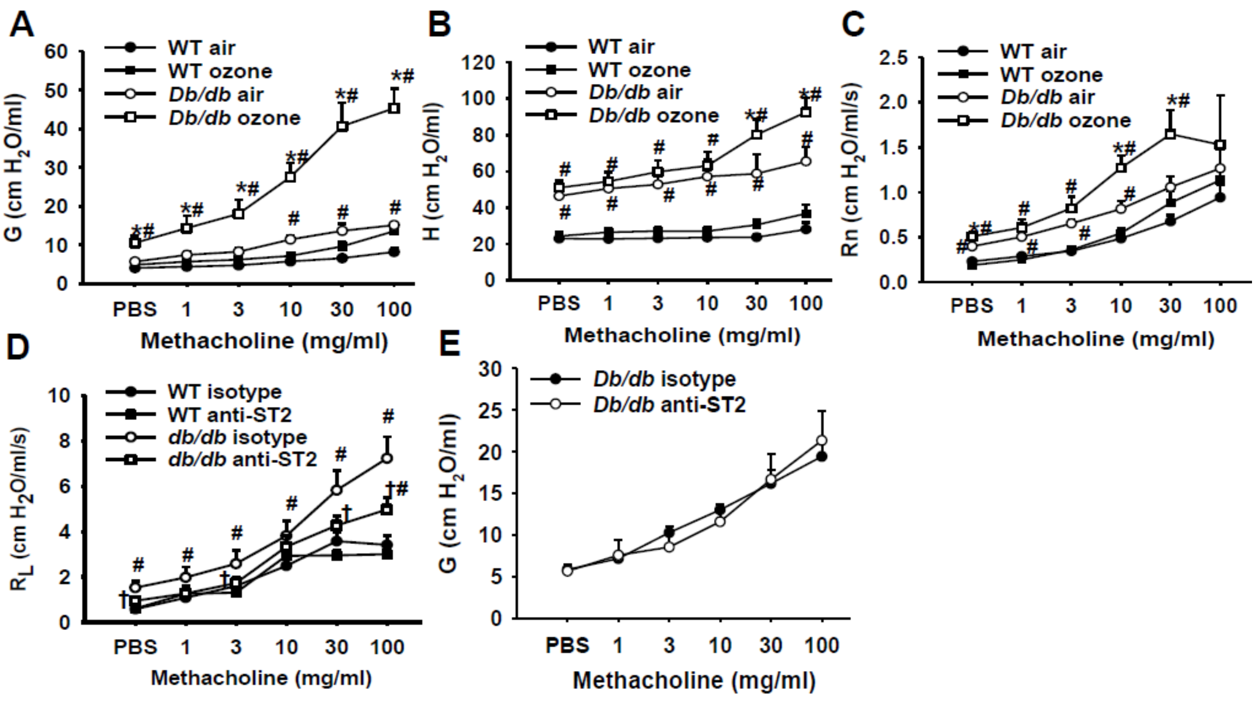

Figure S1. O_3-induced AHR is augmented in obese mice and reduced by anti-ST2. Changes in in the coefficients of A) lung tissue damping (G) and B) elastance (H) and in C) Newtonian resistance (R_n) induced by inhaled aerosolized methacholine in lean wildtype (WT) and obese *db/db* female mice exposed to air or ozone (O_3) (2 ppm for 3 h) and studied 24 h after exposure. D) Changes in pulmonary resistance (R_L) in *db/db* and WT mice treated with isotype or anti-ST2 antibody prior to O_3 exposure. E) Methacholine-induced changes in G in *db/db* mice treated with either anti-ST2 or isotype antibody and exposed to air. Results are mean $\pm$ SE of 4-8 mice/group. * $p<0.05$ versus air; # $p<0.05$ versus lean mice with same exposure; † $p<0.05$ versus isotype-treated mice of same genotype

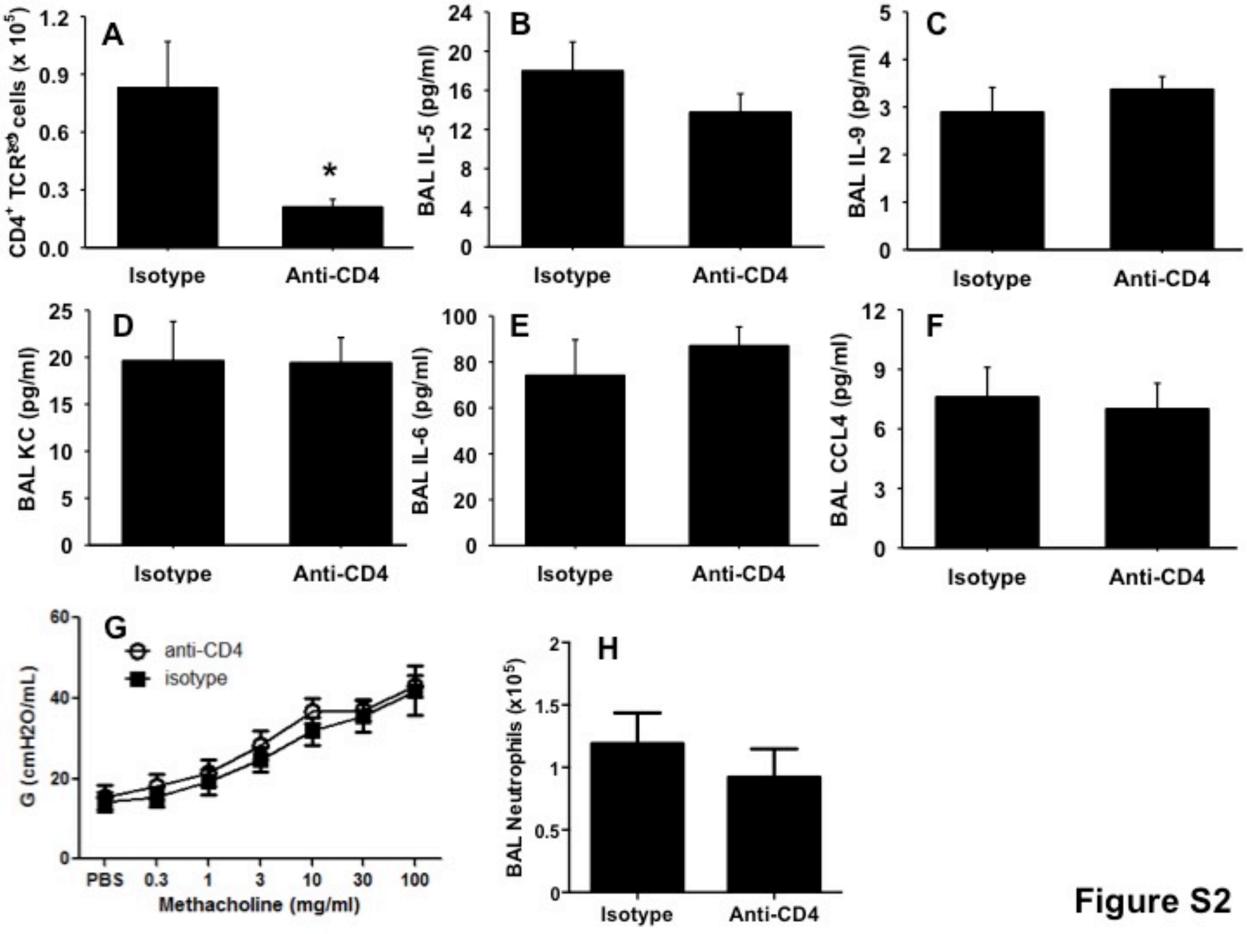

Figure S2. Effect of anti-CD4 on BAL cytokines in obese-O_3 exposed mice. Changes in total lung CD4+ cells (A), and in BAL IL-5 (B), IL-9 (C), KC (D), IL-6 (E), CCL4 (F), Airway hyperresponsiveness (G) and BAL neutrophils (H) in *db/db* mice treated with anti-CD4 or isotype antibody prior to O_3 exposure. Results are mean $\pm$ SE of 4-6 mice/group studied over 4 experimental days. * $p<0.05$ versus isotype

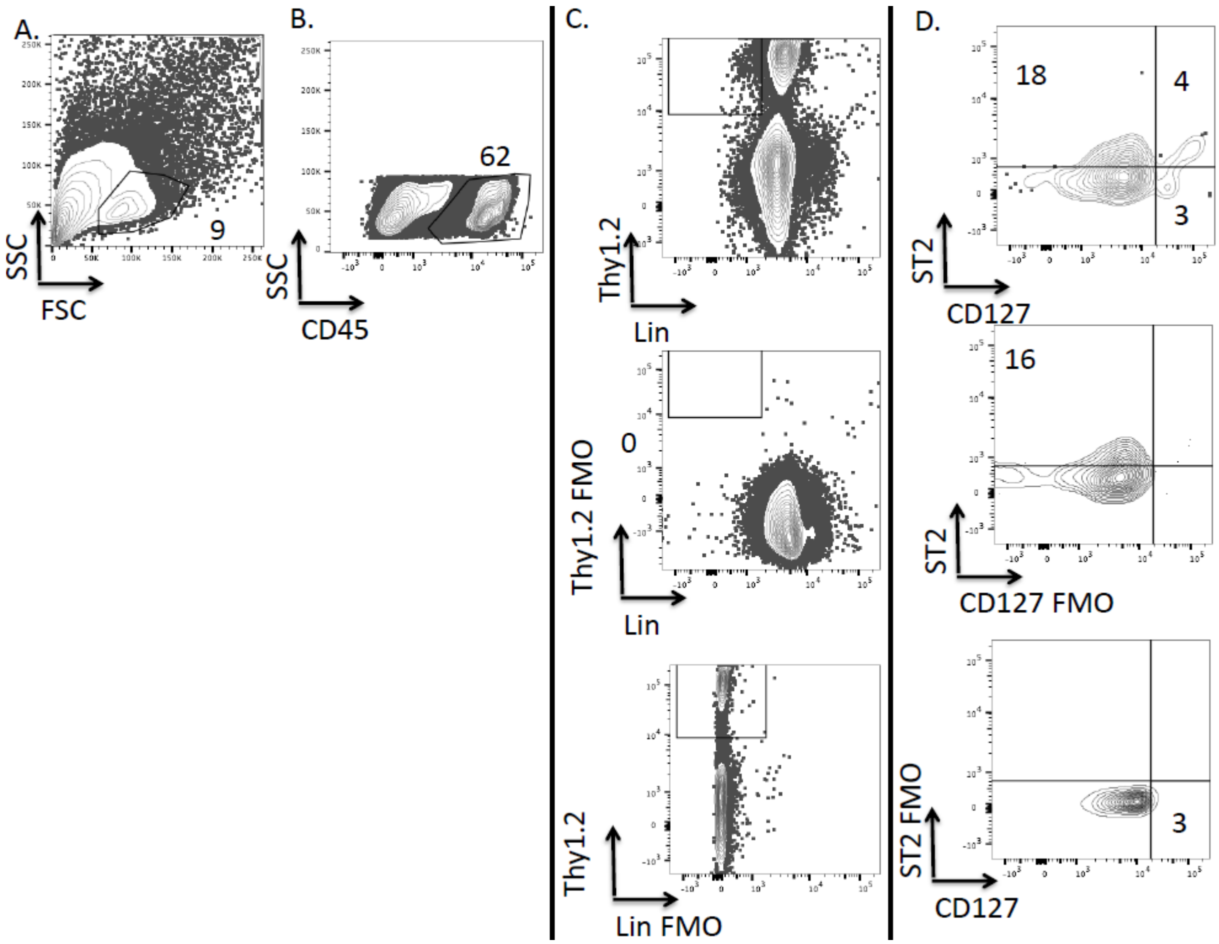

Figure S3. Gating of ILC2. Lungs were disrupted, and single cell suspensions generated. Unstimulated cells were stained with CD45, Thy1.2, a linage cocktail, ST2, and CD127. In A-D are shown stains from one representative *db/db* mouse exposed to O_3. The fluorescence minus one (FMO) control stains for Lin, Thy1.2, ST2, and CD127 are also shown. For control staining, cells from three *db/db* O_3-exposed mice were combined. Total ILC2 (Figure 4A) were gated as $SSC^{Low}FSC^{Low}CD45^{+}Lin^{-}Thy1.2^{+}ST2^{+}CD127^{+}$.

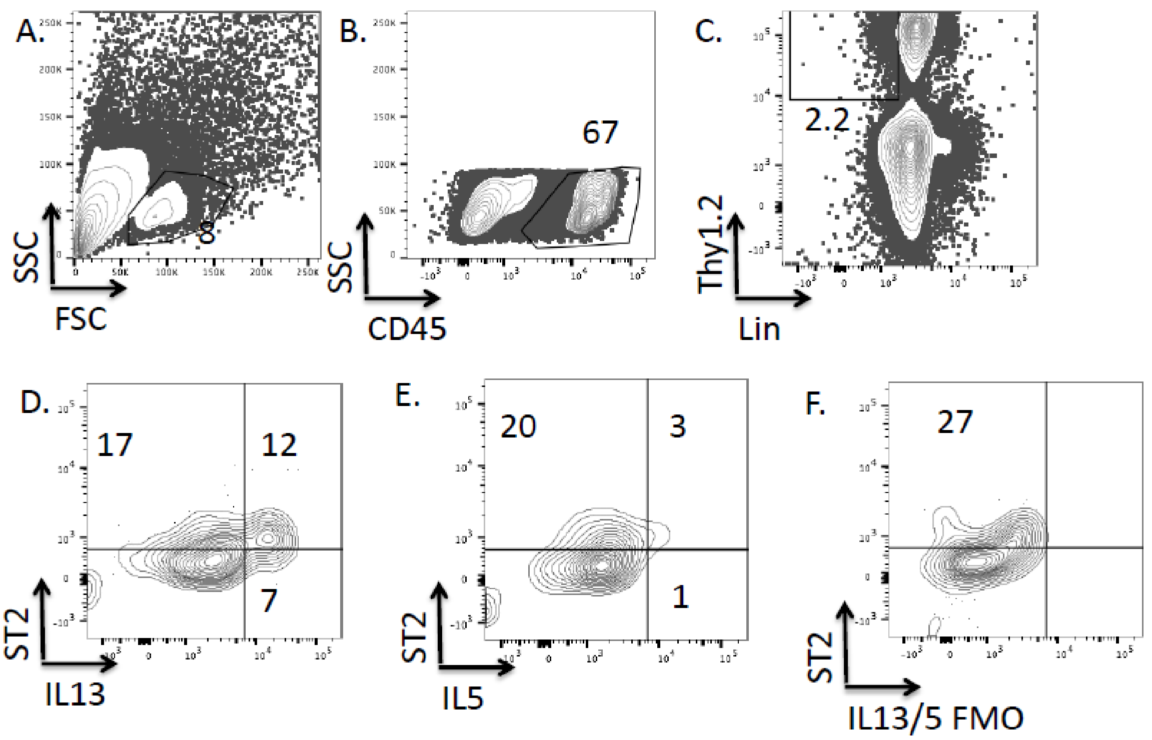

Figure S4. Gating for IL-13$^+$ and IL-5$^+$ ILC2. Lungs were disrupted, single cell suspensions generated, and cells stimulated with PMA and ionomycin as described in Supplemental Methods. Shown is a representative stain from one *db/db* O_3-exposed mouse. Cells were stained with antibodies to CD45, Thy1.2, a linage (Lin) cocktail, IL-5 or IL-13, and ST2. IL-13$^+$ and IL-5$^+$ ILC2s were gated as SSC^{Low}FSC^{Low}CD45$^+$Lin$^-$Thy1.2$^+$IL-13$^+$ and SSC^{Low}FSC^{Low}CD45$^+$Lin$^-$Thy1.2$^+$IL-5$^+$ respectively. As confirmation of the cells ILC2 state, cells were also stained with ST2, shown in the upper right quadrant are those cells that are SSC^{Low}FSC^{Low}CD45$^+$Lin$^-$Thy1.2$^+$IL-13$^+$ST2^{High} or SSC^{Low}FSC^{Low}CD45$^+$Lin$^-$Thy1.2$^+$IL-5$^+$ST2^{High} which the majority were. The fluorescence minus one (FMO) control stains for IL-13 and IL-5 (which used the same isotype) are also shown. For control staining, from three *db/db* O_3-exposed mice were combined.

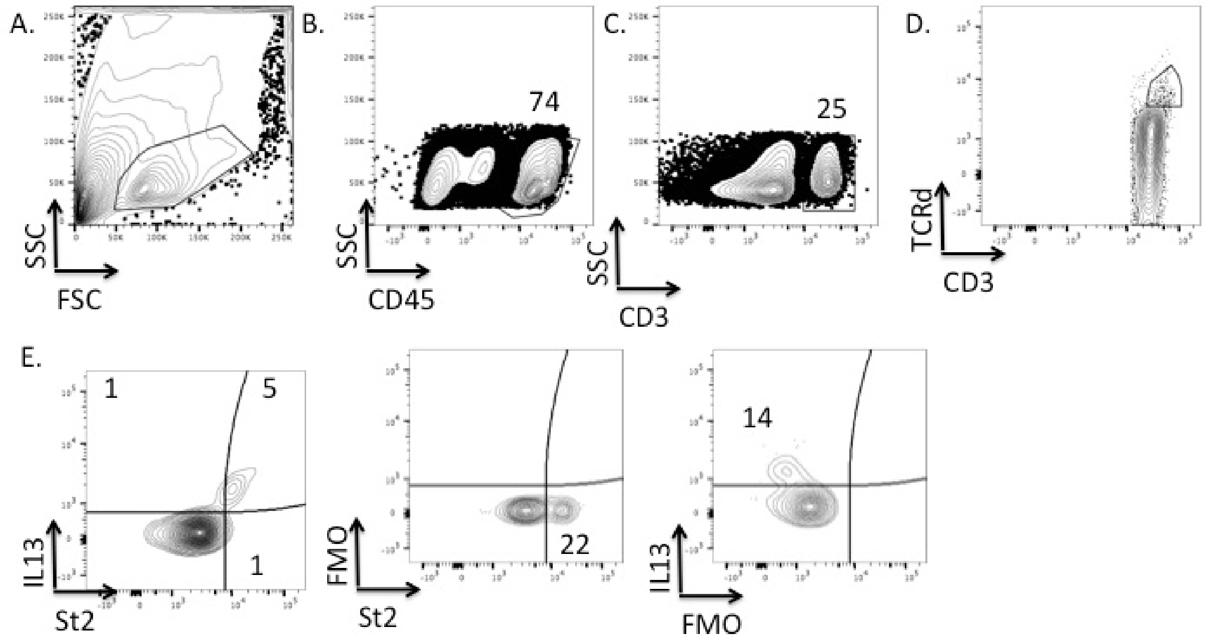

Figure S5. IL-13$^+$ST2$^+$ costaining on γδ T cells. Lungs were disrupted, single cell suspensions generated, and cells stimulated with PMA and ionomycin as described in Supplemental Methods. Cells were stained with antibodies to CD45, CD3, TCRδ, IL-13, and ST2. IL-13$^+$ γδ T cells (Figure 4C,E) were gated as SSC^{low}CD45$^+$CD3$^+$TCRδ$^+$IL-13$^+$ and ST2$^+$γδ T cells were gated as SSC^{low}TCRδ$^+$ST2$^+$ (Figure 4C,D). To determine if the IL-13$^+$ γδ T cells were also positive for ST2, cells were gated as SSC^{low}CD45$^+$CD3$^+$TCRδ$^+$IL-13$^+$ST2$^+$. Shown is one representative stain from a *db/db* mouse exposed to O_3. For the fluorescence minus one (FMO) control stains, cells from three *db/db* O_3-exposed mice were combined.
